# Paircomp, FamilyRelationsII and Cartwheel: tools for interspecific sequence comparison

**DOI:** 10.1186/1471-2105-6-70

**Published:** 2005-03-24

**Authors:** C Titus Brown, Yuan Xie, Eric H Davidson, R Andrew Cameron

**Affiliations:** 1 Division of Biological Sciences, California Institute of Technology, Pasadena, CA 91125, USA; 2 Center for Computational Regulatory Genomics, California Institute of Technology, Pasadena, CA 91125, USA

## Abstract

**Background:**

Comparative sequence analysis is an effective and increasingly common way to identify *cis*-regulatory regions in animal genomes.

**Results:**

We describe three tools for comparative analysis of pairs of BAC-sized genomic regions. Paircomp is a tool that does windowed (ungapped) comparisons of two sequences and reports all matches above a set threshold. FamilyRelationsII is a graphical viewer for comparisons that enables interactive exploration of several different kinds of comparisons. Cartwheel is a Web site and compute-cluster management system used to execute and store comparisons for display by FamilyRelationsII. These tools are specialized for the discovery of *cis*-regulatory regions in animal genomes. All tools and their source code are freely available at .

**Conclusion:**

These tools have been shown to effectively identify regulatory regions in echinoderms, mammals, and nematodes.

## Background

Comparative sequence analysis is fast becoming a standard method for discovering *cis*-regulatory modules [1]. The technique relies on the signatures of conservation left by functional genomic regions as the background sequence evolves. It is often the only way to computationally discover *cis*-regulatory modules in animal genomes when definite knowledge of upstream regulators is lacking, and it can serve as an excellent complement to experimental techniques.

Paircomp, FamilyRelationsII (FRII), and Cartwheel are an integrated system for comparing two BAC-sized (~100 kb) genomic sequences, viewing the comparison, manipulating thresholds and views, and extracting the results. These tools and their predecessors, seqcomp and FamilyRelations, have been used extensively in the years since we first made them available [2]. However, the addition of Cartwheel, a Web server system for performing, storing, and revisiting analyses, makes this combined toolkit considerably more useful to the experimental biologist.

The first analysis done with FamilyRelations was a comparison of the *otx *region between two sea urchins; 11 of the 17 conserved blocks were shown to drive expression of a reporter [3]. Kirouac and Sternberg [4] showed that features conserved between *C. elegans *and *C. briggsae *encode functional regulatory regions. Romano and Wray [5] used FamilyRelations to show that primary sequence identity was conserved in only part of the previously identified *endo16 cis*-regulatory region, when the *L. variegatus *sequence was used as a partner to the *S. purpuratus *sequence. Leung *et al. *[6] used FRII to analyze regions in which NFKB bound to verify that the regions were conserved between mouse and human. And, most recently, Revilla-i-Domingo *et al. *[7] identified a small conserved region in the *delta *genomic locus as a *cis*-regulatory element responsible for localized expression of *delta *in *S. purpuratus*. Similar analyses of the regulation of *gatae*, *krox*, *wnt8*, *brachyury*, *tbrain*, *foxa *and *deadringer *in *S. purpuratus *are forthcoming from this lab. While most published use of FRII and Cartwheel has been in sea urchins and nematodes, users have reported that the tools accurately identify regulatory regions in vertebrates and plants.

FRII and Cartwheel are specialized for identifying conservation within relatively small genomic regions, and can be used for comparing BAC sequences between organisms for which no whole genome assembly exists (e.g. *S. purpuratus/L. variegatus*). The exhaustive "dot-plot"-style search algorithm used (described below) assumes nothing about the relative positioning or orientation of regulatory regions and can be used to detect rearrangements that might be missed by a global alignment algorithm (see e.g. [4]). Because of these features, FRII and Cartwheel are particularly useful in targeted searches for regulatory regions.

In this paper, we present these effective tools for comparative sequence analysis to the wider biological community.

## Implementation

Paircomp is a program for doing windowed comparisons of two sequences. It is an expanded reimplementation of the seqcomp program [2]. Paircomp contains several algorithms for doing exhaustive fixed-width-window sequence comparisons, optimized for different parameters. The default algorithm uses a sliding window to do a "rolling comparison" and runs in time O(NxM) for two sequences of lengths N and M. Paircomp is written in C++ and has a Python interface.

FamilyRelationsII (FRII) is a graphical viewer for sequence analyses. It is a C++ reimplementation of the original Java/Jython FamilyRelations [2]. FRII uses the cross-platform FLTK windowing toolkit to present a common interface on Windows, Mac OS X, and Linux/X11.

Cartwheel is a server-side system that presents a uniform interface for job coordination and execution. It has several components, including a Web interface through which users can establish analyses; a remote interface for programs to retrieve analysis data; and a batch job queueing system based on a method of parallel processing known as a Linda tuple space. All of the components are built on top of a PostgreSQL database. Cartwheel is written in Python and provides libraries in Python, Java, and C++ for remote access.

A technical history of the design decisions made in the implementation of these tools has been published online ([8], article "Python in Bioinformatics").

### Availability

FRII is freely available for download in a binary distribution for Mac OS X and Windows [9]; FRII will also run under most UNIX distributions but must be compiled individually. The Center for Computational Regulatory Genomics at Caltech maintains a public Cartwheel server [10]. A tutorial for FRII is available online [11], and an example homework assignment for an undergraduate class is also available. The source code for paircomp, FRII and Cartwheel and all their components is freely available under the L/GPL through the above Web sites. Paircomp, FamilyRelationsII and Cartwheel are Copyright ^© ^2001–2004 the California Institute of Technology.

## Results and discussion

### Paircomp

Several different classes of algorithms are available for comparing two genomic sequences. Windowed comparisons do an exhaustive comparison of two sequences with a fixed-width window, and record strict (ungapped) sequence identity within that window [2,12]. Local alignment algorithms such as BLAST search for common "words" of DNA in a pair of sequences and build a gapped alignment around these words [13]. These gapped alignments are often scored by overall length, so that e.g. a 500 bp match at 90% is ranked higher than a 200 bp match at 90%. Global alignment algorithms such as AVID [14] and LAGAN [15] seek to build a start-to-end gapped alignment of syntenic genomic regions. Windowed comparisons and local alignment algorithms usually search for matches in both forward and reverse complement directions, while global alignment algorithms typically try to build an alignment without inversions. Implementations of all three strategies for genomic comparisons have been publicly available for some time: Dotter and seqcomp implement windowed comparisons [2,12]; PipMaker uses a local alignment algorithm, blastz [16,17]; and Vista relies on a global alignment generated by AVID [18]. All three comparison strategies have been successful at finding regulatory regions [1,19].

Of the three general classes of algorithms, we chose to use windowed comparisons in our search for *cis*-regulatory modules. Our decision was based on several criteria. First, these comparisons report matches based solely on strict sequence identity with no gapping, unlike alignment algorithms. This is a good *ab initio *requirement when comparing sequences in search of *cis*-regulatory modules, whose evolution is still poorly understood; in particular, binding sites could be sensitive to indels, which are somewhat elided in gapped alignments. Moreover, we had no *a priori *expectation for the locations, sizes, or degrees of similarity of conserved regions, necessitating an exhaustive search strategy that did not bias scores based on the length or position of matches. And, finally, from a user-interface perspective the parameters for paircomp – windowsize and threshold – are simple and intuitively linked to the results. Our success with this basic approach means that we have not needed to move to alternative algorithms.

Paircomp is a standalone program that executes windowed comparisons (see Methods). It searches for matches in both the forward and reverse complement directions. Paircomp runs within Cartwheel; the results are stored in a database and communicated to FRII.

### Cartwheel

Cartwheel is a Web site through which analyses are executed and from which analyses are loaded into FamilyRelationsII. It provides an easy-to-use interface through which to establish a set of analyses on a pair of sequences. Cartwheel also allows the annotation of sequences with a variety of features; features can be uploaded to Cartwheel in the standard GFF format. A tutorial for setting up pairwise comparisons is available online [11].

### FamilyRelationsII

FamilyRelationsII, or FRII, displays comparisons of BAC-sized genomic sequences of lengths ~100 kb. It is a graphical program that runs directly from a desktop and loads data from the Cartwheel server. From within FRII, users can zoom in to look more closely at features, alter scoring thresholds for comparisons, change the color of features, and turn on or off the display of specific analyses. FRII can also display closeup views of comparisons and alignments against DNA and protein sequence.

**Figure 1:**
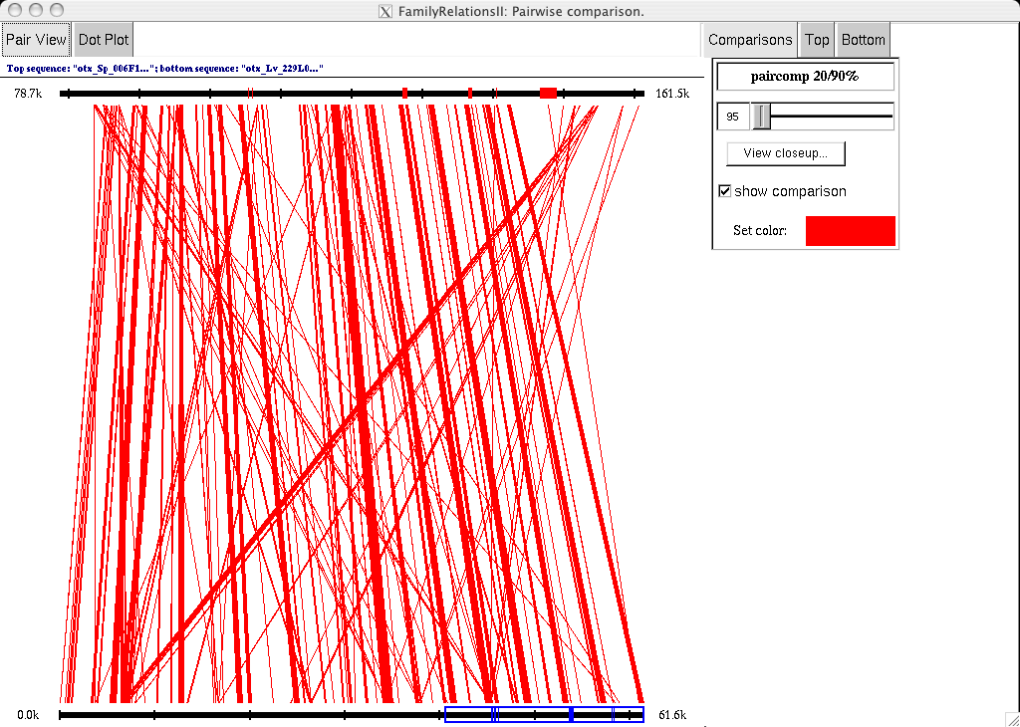
A paircomp comparison of the *otx *gene locus from *S. purpuratus *(top) with *L. variegatus *(bottom). We used paircomp to compare all 20 bp subsequences from a 160 kb *S. purpuratus *BAC with a 62 kb *L. variegatus *BAC; those 20 bp subsequences with a exact match of 19/20 or 20/20 bases are connected with a red line. Only the 80 kb surrounding the *otx *gene is shown on the top. Matches to the known *S. purpuratus *cDNA sequence are shown in red on the top sequence, and TBLASTX matches in *L. variegatus *to the same cDNA sequence are shown in blue on the bottom sequence. The *L. variegatus *genomic sequence does not extend to cover the 3' region of the coding sequence. On the top of the view are tabs to switch between the "pair view" (shown) and the "dot plot" view (see Figure 2). On the right side of the view are control buttons that allow the user to change both the color and the threshold at which matches are displayed. The user can also view a closeup of a region by selecting the region on the sequence (e.g. as on the bottom sequence, where a region from 40 kb to 61.6 kb is selected) and then pressing the "View closeup..." button. An example closeup view is shown in Figure 3.

Figure 1 shows the main FRII view of a comparison between the *otx *locus in *S. purpuratus *and *L. variegatus*, two sea urchins that diverged approx. 50 mya. The genomic sequences were obtained from BAC libraries as described in [3]. In the case of *S. purpuratus*, the BAC contains the entire *otx *coding region; the *L. variegatus *sequence contains only the 5' region of the gene, and not the final exon.

**Figure 2:**
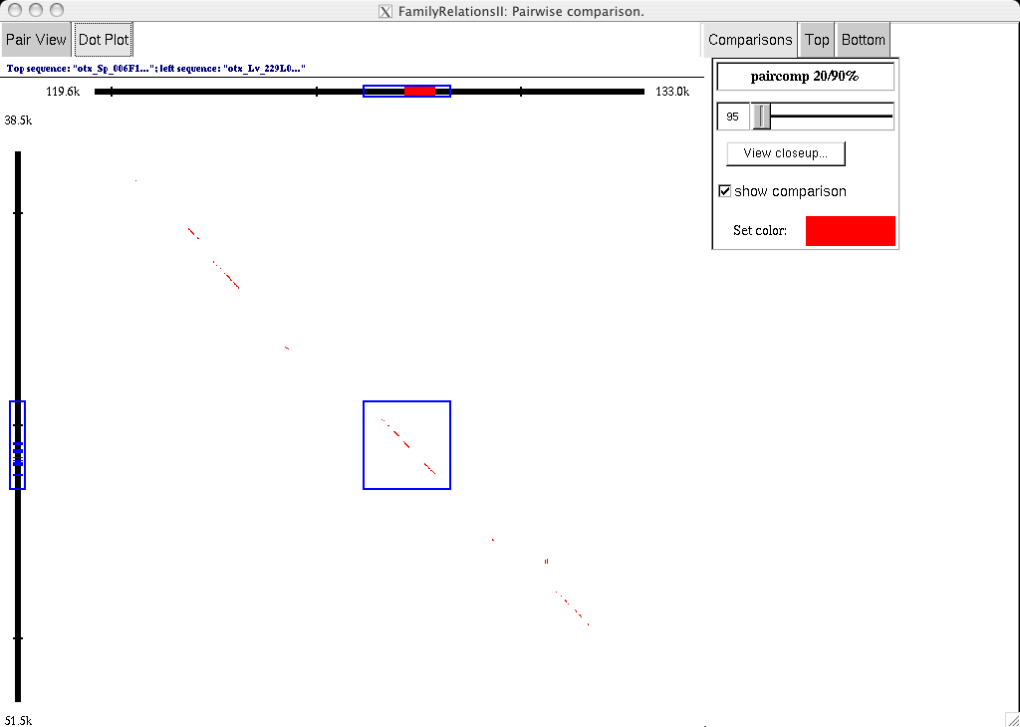
A "dot-plot" style view of a subregion of the *otx *comparison (see Figure 1). The top sequence is a zoomed-in view of the *otx *genomic region from *S. purpuratus*, as in Figure 1; the region runs from 119.6 kb to 133.0 kb. The side sequence is a zoomed-in view of the orthologous region from *L. variegatus*, running from 38.5 kb to 51.5 kb. The region surrounding the first exon (in red) of the *sp α-otx *transcript is selected on the top (*S. purpuratus*) sequence, and the corresponding TBLASTX matches are highlighted on the left (*L. variegatus*) sequence in blue. The selection box in the center of the view contains the paircomp matches in this region, showing only 20 bp matches that match at 19/20 or 20/20 (corresponding to a 95% threshold). A closeup view of this region, showing the DNA sequence of the two regions with the corresponding matches, is shown in Figure 3.

The comparison shown is a paircomp comparison performed with a 20 bp window at 90% and then displayed at a 95% threshold. The general colinearity of the matches suggests that the majority of the similar regions are conserved with respect to size, orientation, and relative distance from the exons. This colinearity is typical of conserved features in our comparisons. The diagonal lines crossing the comparison often identify low complexity regions such as simple sequence repeats present throughout both genomic regions. This pairwise mapping view is one of the two large-scale views in FRII; the other large-scale view is a dot-plot view, shown in Figure 2.

Figure 2 shows a dot-plot view of an expanded region of the comparison, centered on the first exon of the *α-otx *transcript. In addition to the exon itself, there is patchy conservation throughout the region; again, this is typical of many comparisons. This view also shows that all of the elements are collinear on scales of ~10 kb.

In both the dot-plot and pairwise mapping view, multiple comparisons done with different parameters can be displayed in different colors. The threshold for the matches shown can be adjusted until the desired view is obtained, and sequence can be exported from any of the views via a pop-up menu.

**Figure 3:**
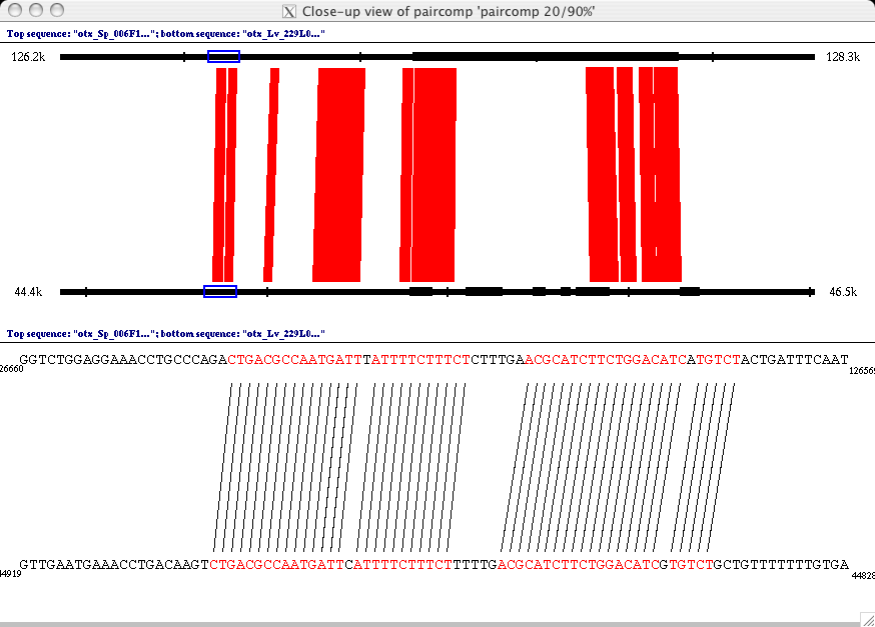
A closeup view of the paircomp comparison of the genomic sequence surrounding the first exon of *otx *in *S. purpuratus *(top sequence) and *L. variegatus *(bottom sequence). The top half of the closeup view shows orthologous 2 kb genomic regions (126.2 kb – 128.3 kb in the *S. purpuratus *BAC, 44.4 kb – 46.5 kb in the *L. variegatus *BAC). Matches of 19/20 or 20/20 bases are drawn in red between the sequences, and the exon matches from Figure 2 are shown in black on the sequence lines. The bottom half of the closeup view shows the part of the sequence selected in blue on the top half of the view. Lines are drawn in black between individual matching bases, and the matching bases are colored in red. Note that both blocks shown match at 19/20 because of the single mismatch in the middle of the blocks.

Once a threshold is chosen, the user can expand the view of a particular region. Figure 3 shows a closeup view of the region outlined in blue in Figure 2. The sequence shown in Figure 3 is a small patch of conservation upstream of the first exon, displayed at a 19/20 threshold. Here the user scans along the sequence and visually compares both the boundaries of the matches and the complexity of the sequence. Sequences are directly exported to other applications via the "paste" buffer.

FRII also performs searches for motifs using the IUPAC notation in which e.g. W represents A or T. This feature allows users to search for matches to known "consensus" binding sites for transcription factors. Searches are either stored on the Cartwheel server and displayed as individual features on FRII views, or executed directly in FRII. One particularly convenient feature is the ability to ask for motifs that have mismatches in up to 5 positions; this lets users search for weaker matches to known consensi.

### Other analyses

FRII displays a variety of analyses. In addition to paircomp windowed comparisons, FRII displays and manipulates Vista-style comparisons, BLAST and blastz comparisons, BLAST database searches, cDNA and protein comparisons, and the results of several different gene finders (genscan, geneid, and hmmgene [20-22]). All of these analyses may be executed directly on the Cartwheel server, excepting only Vista comparisons using the (default) AVID alignment program. The data for Vista comparisons must be uploaded from the results returned by the Vista Web site; however, Vista-style comparisons with the LAGAN global alignment tool are executed directly on Cartwheel.

### Discovering and analyzing regulatory regions

We and others have successfully used paircomp, FRII, and Cartwheel to discover a number of regulatory regions (see Introduction). Once we have a pair of genomic regions to compare, the steps we follow are essentially invariant from region to region:

1. We set up two to three paircomp analyses at the following windowsizes and thresholds: 10 bp/90%; 20 bp/80%; 50 bp/60%.

2. We match the cDNA or protein of interest against both regions, to determine where the coding regions lie.

3. We also compare the RefSeq database from NCBI against both regions, to find other genes in the region.

4. We load these analyses into FRII and zoom in to a view that includes as much intergenic sequence around the gene as is possible without also including other genes. We then adjust the thresholds on the 20 bp and 50 bp analyses until we obtain a roughly collinear pattern of conserved blocks. Typical values for these thresholds are 80–100% for a 20 bp windowed comparison, and 60–80% for a 50 bp windowed comparison.

5. We use the closeup view to extract the conserved blocks, and design PCR primers to isolate all of the contiguous blocks of conserved sequence. We then individually subclone or fuse them into a GFP reporter construct together with a basal promoter. These constructs are then introduced into the sea urchin by microinjection and analyzed for appropriate spatiotemporal expression.

In our experience, we have always been able to identify the relevant enhancer elements using this procedure. A similar procedure in which putatively negative elements are fused with a ubiquitous driver of expression often identifies necessary repressive elements. Also note that one caveat of these procedures is that for some genes, e.g. transcription factors, there are often many regions that appear to do nothing. These may be regulatory regions that affect expression at times or in places that are not under consideration, or could be other genomic features not relevant to gene regulation.

## Conclusion

Paircomp, FamilyRelationsII, and Cartwheel are an effective, easy-to-use set of tools for analyzing conservation in BAC-sized genomic regions. Over 100 people are currently using them, and they have been effective in finding regulatory regions in a variety of organisms. In this paper we have described the tools and provided an introduction for biologists who wish to use them.

## Availability and requirements

See Implementation, above, for information on server-side software.

**Project name: **FamilyRelationsII

**Project home page: **

**Operating systems: **Mac OS X, Windows NT/XP, UNIX/Linux (X Windows)

**Programming language: **C++

**License: **GPL/LGPL

No restrictions placed on use.

## Authors' contributions

CTB designed and implemented the majority of the functionality described. YX implemented a significant portion of the XML-RPC functionality used for client-server interaction. EHD laid out the design requirements, aided in writing the paper, and supervised the development of FRII. RAC is responsible for running the servers and did the majority of bug testing, and also contributed to the paper.
